# Computational Psychiatry: towards a mathematically informed understanding of mental illness

**DOI:** 10.1136/jnnp-2015-310737

**Published:** 2015-07-08

**Authors:** Rick A Adams, Quentin J M Huys, Jonathan P Roiser

**Affiliations:** 1 Institute of Cognitive Neuroscience, University College London, London, UK; 2 Division of Psychiatry, University College London, London, UK; 3 Translational Neuromodeling Unit, University of Zürich and Swiss Federal Institute of Technology, Zürich, Zürich, Switzerland; 4 Department of Psychiatry, Psychotherapy and Psychosomatics, Hospital of Psychiatry, University of Zürich, Zürich, Switzerland

**Keywords:** SCHIZOPHRENIA, DEPRESSION, PSYCHIATRY, COGNITION, PSYCHOPHARMACOLOGY

## Abstract

Computational Psychiatry aims to describe the relationship between the brain's neurobiology, its environment and mental symptoms in computational terms. In so doing, it may improve psychiatric classification and the diagnosis and treatment of mental illness. It can unite many levels of description in a mechanistic and rigorous fashion, while avoiding biological reductionism and artificial categorisation. We describe how computational models of cognition can infer the current state of the environment and weigh up future actions, and how these models provide new perspectives on two example disorders, depression and schizophrenia. Reinforcement learning describes how the brain can choose and value courses of actions according to their long-term future value. Some depressive symptoms may result from aberrant valuations, which could arise from prior beliefs about the loss of agency (‘helplessness’), or from an inability to inhibit the mental exploration of aversive events. Predictive coding explains how the brain might perform Bayesian inference about the state of its environment by combining sensory data with prior beliefs, each weighted according to their certainty (or precision). Several cortical abnormalities in schizophrenia might reduce precision at higher levels of the inferential hierarchy, biasing inference towards sensory data and away from prior beliefs. We discuss whether striatal hyperdopaminergia might have an adaptive function in this context, and also how reinforcement learning and incentive salience models may shed light on the disorder. Finally, we review some of Computational Psychiatry's applications to neurological disorders, such as Parkinson's disease, and some pitfalls to avoid when applying its methods.

## Introduction

Computational Psychiatry aims first to model the computations that the brain performs—that is, the brain's solutions to the problems it faces—and second to thereby understand how the ‘abnormal’ perceptions, thoughts and behaviours that are currently used to define psychiatric disorders relate to normal function and neural processes. By formalising mathematically the relationship between symptoms, environments and neurobiology, it hopes to provide tools to identify the causes of particular symptoms in individual patients.

Computational Psychiatry is at least partially motivated by the shortcomings of the current psychiatric classification systems (the Diagnostic and Statistical Manual of Mental Disorders, or DSM-5,^1^ and the International Classification of Diseases, or ICD-10^2^), in which the symptoms entail the diagnosis and which lack mechanistic explanations for mental symptoms. The reliability of diagnostic systems was ‘bought at the price of validity’^3^—meaning clinicians have some confidence that given a set of symptoms they would all make a consistent diagnosis, but no confidence that that diagnosis corresponds to a single biological or psychological entity, or that it can predict the outcome of either the illness or a given treatment. Likewise, the biopsychosocial model of mental illness^4^ has had great success in helping clinicians understand illness at a human level, but as a causal account it fails: its constituent parts (particularly the biological and psychosocial) are separated by a wide explanatory gap.

The National Institute of Mental Health (NIMH) generated the Research Domain Criteria (RDoC^5^) in an attempt to revive psychiatric classification with a bracing dose of mechanistic validity. The RDoC consists of five ‘domains’ of mental functions that are each described at multiple levels or ‘units of analysis’: the hope is that these units will yield biomarkers to distinguish normal and abnormal functioning. In principle, this approach has several advantages, but we note that the current RDoC (it is a working document) views psychiatric disorder—including its social risk factors—through a very biological lens. Indeed, its units of analysis step from ‘genes’ to ‘molecules’ to ‘cells’ to ‘circuits’ to ‘physiology’, and then leap straight to ‘behaviour’. Computational Psychiatry provides some of the tools to link these levels.

Numerous authoritative reviews of initial developments in Computational Psychiatry already exist,^6–15^ alongside pioneering work by Hoffman,^16^ Cohen^17^ and many others. In this article, we look towards the future and—using examples from depression and schizophrenia—illustrate Computational Psychiatry's potential for reconceptualising psychiatric disorders and generating new hypotheses. Prior to this, we briefly rehearse the advantages in adopting a Computational Psychiatry approach.

### Computational Psychiatry unites many levels of description

Computational Psychiatry's organising principles arose in computational neuroscience, when Marr^18^ identified three levels at which the problems solved by the brain may be described. At a ‘computational’ level, the formal nature of the problem has to be described: What are the mathematical and statistical issues involved? What solutions do these issues allow? The ‘algorithmic’ level describes the method of solving the problem. This may be an approximation or a much more complex procedure. The ‘implementational’ level describes the physical realisation of this method: How does coordinated activity in neurons or brain circuits encode these algorithms?

Critically, these three levels are not entirely independent. Although any algorithm could be implemented physiologically in many ways, constraints at one level have implications at other levels: Some computations (eg, high-dimensional integrals) may be very laborious for neural systems, so algorithmic approximations become necessary. System failures caused by complex computational problems can therefore provide important clues about underlying algorithms.

In the biological sphere, this simple trinity is crossed with other relevant levels of description. For example, the implementational level itself can be decomposed into RDoC's various ‘units of analysis’, from genes to physiology as well as (we would argue) social interaction.^8^ ^19^ With respect to most mental disorders, Computational Psychiatry lies at the nexus of these descriptive levels and makes them explicit.

### Computational Psychiatry is mechanistic and rigorous

Computational Psychiatry is mechanistic in a way that the DSM-5, ICD-10 and biopsychosocial model can never be, thanks to its use of generative models. A generative model is a probabilistic description of how high-level causes actually generate low-level data (in contrast, a discriminative model merely describes how to label such data with their likely causes^i^). This distinction is important because knowing how causes generate data allows a model to generate synthetic or ‘simulated’ data from given causes.

**Figure 1:**
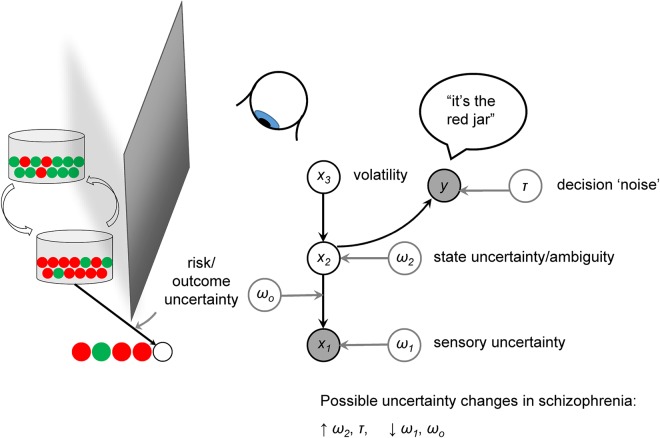
A hierarchical generative model, illustrated using the ‘beads’ or ‘urn’ task. On the left, two jars are hidden behind a screen, one containing mostly green and some red balls, the other the converse. A sequence of balls is being drawn from one of these jars, in view of an observer, who is asked to guess from which jar they are coming. We have illustrated a simple hierarchical generative model of this process on the right: the observer is using such a model to make his/her guess. Variables in shaded circles are observed, and variables in unshaded circles are ‘hidden’ (ie, part of the model only). At the bottom of the model is x_1_, the colour of the currently observed bead. Uncertainty about this quantity (eg, if the light is low or if the participant is colour-blind) is denoted as ω_1_. At the next level of the model is *x_2_*, the belief about the identity of the current jar, and its associated uncertainty ω_2_, known as state uncertainty or ambiguity. Another form of uncertainty, risk or outcome uncertainty (ω_0_) governs the relationship between the identity of the jar and the next outcome: Even if we are sure of the jar's identity, we cannot be certain of the colour of the next bead. At the top of the model is the belief about the probability that the jars could be swapped at any time, known as volatility. We have not shown them here but this could have its own associated uncertainty, and there could be further levels above this. Last, the participant must use his/her belief about the identity of the jar to make a guess: The mapping between this belief and the response *y* is affected by a degree of stochasticity or decision ‘noise’, τ. In schizophrenia, there may be too much uncertainty (ie, lower precision) in higher hierarchical areas that encode states or make decisions, and an underestimation of uncertainty in lower (sensory) areas.

This generative description can be of how brain activity generates brain imaging data, or of how states in the world evolve and affect an agent's decision-making (eg, figure 1, described in the next section); in the latter case, commonly used in Computational Psychiatry, we are modelling the brain's own model of the world.^20^ By altering key parameters in our generative models of agents’ brains, we can observe what effects they have on decision-making and use this information to optimise experimental design or make counter-intuitive predictions. Bayesian statistics and machine learning techniques then allow this entire description to be tested against real data for goodness-of-fit. Comparisons of generative models by means of Bayesian model selection offer among the most rigorous and global comparative assessment of scientific hypotheses.^21^

Formulating a generative model requires an explicit description of the mathematical details of the cognitive or neural process from the outset. This is difficult but important, as it can force one not only to think hard about what particular constructs (such as ‘attention’ or ‘salience’) really mean,^22^ but also to be very explicit about assumptions and ignorance.

### Computational Psychiatry is not biologically reductionist

Computational Psychiatry is of course reductionist in the sense that it wishes to reduce cognitive processes to computations. Importantly, however, it does not view genes, neurotransmitters or neural circuits as causes of mental illness separate to the context in which the agent operates.^23^ Indeed, it is precisely the agent's environment (both physical and social) which the nervous system may be trying to model, and which our models must also reflect.^8^ ^19^

It is true that the new science of epigenetics (heralded by Engel^4^ almost 40 years ago) also places genes firmly in their environmental context; but as an explanation of mental illness, a gene–environment interaction in the absence of any computational specification is a sandwich without the meat. What Psychiatry ultimately wants to know is: How and why does this gene–environment interaction change inference (and thereby experience and behaviour)?

### Computational Psychiatry is not artificially categorical

One important, but unfulfilled, aspiration of the architects of DSM-5 was to move beyond purely categorical diagnoses to a more dimensional system, as it seems our current categories are not valid at the clinical^24^ or genetic^25^ levels. Such an approach would not classify a person with psychosis as just one of ‘schizophrenic’, ‘bipolar’ or ‘schizoaffective’, but might instead score them on scales of ‘manic’ and ‘depressive’ mood symptoms, ‘positive’ (delusional and hallucinatory) and ‘negative’ (avolitional) psychotic symptoms, and ‘cognitive impairment’.

Computational Psychiatry can accommodate and inform both categorical and dimensional approaches—each driven by data. For example, one might find that depressed participants and controls differ continuously (dimensionally) on a certain parameter derived from a certain computational model (eg, ‘reward prediction error signalling’).^26^ Alternatively, one might find evidence that different models are used by distinct groups (ie, possible categories) to perform the same task, for example, patients with schizophrenia with high or low negative symptoms,^27^ or those with remitted psychosis and controls.^28^ More generally, having defined alternative (eg, categorical vs dimensional) models, Computational Psychiatry allows one to assess the evidence for competing theories formally, for instance using Bayesian model comparison. Identifying computational categories and dimensions in this way ought to improve both psychiatric nosology^29^ and the targeting and monitoring of treatments.

## Theoretical framework

Computational theories of mind often mirror contemporaneous engineering practices, and vice versa. In the 1970s, for example, philosophers of mind took inspiration from the computer's manipulation of symbols according to deterministic syntactic rules, and sought to explain how humans might also think logically.^30^ Recently, computer science has tried to make machines that can learn and make probabilistic inferences using uncertain or incomplete data, as biological agents can. In this section, we briefly introduce some necessary theoretical constructs in probabilistic inference and action selection before discussing some Computational Psychiatry approaches to depression and psychosis.

### Inferring the present

Put as simply as possible, the brain's fundamental computational task is to infer the state of its environment and choose actions on that basis. Unfortunately, neither its sensory data nor its prior knowledge is completely reliable, and so the brain must use both sources of information—taking into account their uncertainty—to perform its task. The optimal combination of uncertain information is given by Bayes’ theorem, in which a ‘prior’ (the initial expectation of the state of the environment) is combined with a ‘likelihood’ (the probability of the sensory input, given that expectation) to compute a ‘posterior’ (an updated estimation of the state of the environment). For simplicity, these probability distributions are often assumed to be of a kind that can be represented by a few ‘sufficient statistics’; for instance, the mean and precision (inverse variance) of a Normal distribution, in this case both prior and likelihood, can be conveniently weighted by their (scalar) precision.

Aside from their inherent uncertainty, the statistics of natural sensory stimulation are also extremely complex. Nevertheless, as a consequence of the hierarchical structure of the environment, they contain patterns: These patterns are easiest to interpret if the brain's prior beliefs respect the hierarchical structure in its sensory data—that is, if they take the form of a hierarchical model. Hierarchical models explain complex patterns of low-level data features in terms of more abstract causes: for example, the shape that describes a collection of pixels, or the climate that describes annual variation in weather. Hierarchical models are particularly important in the face of complex situations, both behavioural and sensory, and allow for highly efficient decompositions that greatly support planning and simplify optimal decision-making.^31–33^

Hierarchical generative models can use predictive coding (or other methods) to predict low-level data by exploiting their high-level descriptions, for example, reconstructing the missing part of an image.^34^ ^35^ In predictive coding, a unit at a given hierarchical level sends messages to one or more units at lower levels which predict their activity; discrepancies between these predictions and the actual input are then passed back up the hierarchy in the form of prediction errors. These prediction errors revise the higher level predictions, and this hierarchical message passing continues in an iterative fashion.

Exactly which predictions ought to be changed in order to explain away a given prediction error is a crucial question for hierarchical models. An approximately Bayesian solution to this problem is to make the biggest updates to the level whose uncertainty is greatest relative to the incoming data at the level below: that is, if you are very uncertain about your beliefs, but your source is very reliable, you ought to change your beliefs a lot.

Say, for example, I am walking at dusk, and I perceive the movement of a bush in my peripheral vision. I might explain this at various hierarchical levels, as (1) the bush did not actually move; it was a trick of the light; (2) the wind was moving the bush; (3) an animal was moving the bush and (4) a man hiding in the bush, intending to rob me, moved it. The conclusion that I draw will be determined by how precise my beliefs (at each level) are that (1) I saw movement; (2) the wind probably did not cause it; (3) there are probably no animals in the vicinity and (4) there is probably no mugger in the vicinity. The most uncertain (least precise) of these beliefs will have to change (assuming, for the sake of argument, that their likelihoods are equivalent), with very distinct consequences for my subsequent behaviour. Put more formally, the uncertainty (inverse precision) at each level helps determine the learning rate at that level, that is, the size of the adjustments that are made to explain new data.^34^

A classic psychology experiment illustrates uncertainty at different levels (see figure 1). Imagine you are shown two jars of beads, one containing 85% green and 15% red beads, the other 85% red and 15% green. The jars are then hidden and a sequence of beads is drawn (with replacement)—GGRGG. You are asked to guess the colour of the next bead. Even if you are quite certain of the identity of the jar (say, green), you will still be only 85% certain that the next bead will be green. This is ‘outcome uncertainty’ or risk. Imagine you see more beads—the total sequence is GGRGGRR. Now you are very uncertain about the identity of the jar. This is ‘state uncertainty’ or ambiguity. Imagine you see a much longer sequence—GGRGGRRRRRGGGRGGGGGGRGGGG. From this, it seems that the jar changes from green (5 draws), to red (5 draws), to green (remaining sequence). Such temporal changes in hidden causes give rise to ‘volatility’;^36^ ^37^ for example, someone surreptitiously switching the jar during the experiment.

Now suppose that although the real proportions are 85% and 15%, a malicious experimenter misleadingly told you that they are 99.9% and 0.1%. From the 25 draw sequence above, you might reasonably conclude that the jars had actually changed eight times—whenever the colour changed. This is what happens when the precision at the bottom of a hierarchical model is too high relative to the precision at the top: Following a sensory prediction error, the model concludes that there must have been a change in the environment (in this simplistic example, the jar), rather than ‘putting it down to chance’.

This precision imbalance might contribute to the well-known ‘jumping to conclusions’ reasoning bias in schizophrenia^38^ (although another cause might be noisy decision-making^28^) and the formation of delusional beliefs themselves, which commonly arise in an atmosphere of vivid sensory experiences and strange coincidences.^14^ We return to the subject of delusions in the Psychosis section.

### Weighing the future

On top of the inference about current stimuli and states, the brain needs to solve a second, orthogonal and complicating problem, which arises from the fact that behaviours have both immediate and future consequences. A brief moment of pleasure can have nasty consequences and, though tempting, might best be avoided. Conversely, pain now might result in even greater pleasures later. Optimal behaviour needs to weigh the future against the present. Even in the rare circumstances where inference about the present is perfectly realisable, the brain therefore faces a second set of uncertainties. As the future is not known, the values of possible actions (their summed future rewards and punishments) have to be somehow estimated.

The field of reinforcement learning (RL) has delineated two fundamentally different ways in which past experience is used to estimate and predict future rewards and punishments: so-called model-based (MB) and model-free (MF) cognition.

In MB or goal-directed cognition, experience is compiled into a (possibly hierarchical) generative model of the world—a mechanistic, causal understanding of the causes and consequences of actions and events. When faced with a particular situation, this model can be searched, and the quality of various behaviours deduced—even if they have never been tried or experienced. As this involves somehow simulating or inferring future possibilities, it can have high computational costs.

In MF or habitual cognition, conversely, the agent does not store information about state transitions (ie, exactly what is likely to happen next if a particular action is performed); instead, the agent merely records how much reinforcement is obtained when a certain state s_t_ is visited at time t or an action a_t_ is taken. The agent then computes the discrepancy between the expected outcome V_t_(s_t_) and the actually obtained outcome r_t_—the prediction error δ_t_=r_t_+V_t_(s_t+1_)−V(s_t_). MF learning adds a fraction ε of the reward prediction errors δ_t_ to the expectations every time the state s_t_ is visited: V_t+1_(s_t_)=V_t_(s_t_)+εδ_t_ and thereby reduces the discrepancy between expected and received reinforcements. Under some conditions, this will reveal the true, consistent set of values that incorporate ‘future’ outcomes to the extent that these have followed past choices.

MF learning is computationally undemanding but slow and inflexible, so it is undermined by sudden changes to the environment or to the valuation of rewards. One example of this is a rat learning how to obtain salty food in a maze: MB learning would create knowledge of the internal structure of the maze and the kind of rewards available within it, whereas MF learning would register a given sequence of left/right turns as being the best thing to do. If the rat's usual route were blocked, or if it were thirsty, then the MF information would not be useful. The MF account can be extended—for example, using hierarchical models—to allow more sensitivity to changes.^39^

We next describe these issues in the context of depression and schizophrenia, with the discussion of depression focusing more on valuation, that is, weighing the future, and that of schizophrenia on inference in the present.

## Valuation in depression

Depression is by its nature an aversive state, usually accompanied by negative thoughts about the self, the world and the future, and sometimes characterised by reduced interest and/or pleasure known as anhedonia. However, the core symptoms of depression—sadness, a lack of energy and a reduced ability to enjoy things—are also a frequent but temporary feature of everyday affective experience. The symptoms can become a condition of potentially life-threatening severity if they become part of a vicious circle of negative affect, cognition and behaviour that is impervious to positive influences.

Computational descriptions of normative and resource-rational choices—RL and Bayesian decision theory—reveal many ways in which such a vicious circle could arise. We review how a stable state of anhedonia might exist in RL, and then close with a few speculative suggestions about the most likely paths to this state. Parts of this are described in more detail elsewhere.^40^ ^41^

### Primary utility

With respect to the potential causes of depression, the most obvious candidate in RL is the so-called utility or reward function r. This function uses one scalar number to describe how rewarding (increasingly positive) or punishing (increasingly negative) events in the world are. Some kinds of events may have genetically encoded reward or punishment utilities—most likely, only those of direct relevance to the individual's genetic fitness. Other events acquire ‘utility’ through experience and inference. A very broad undervaluation as in anhedonia could arise from reductions in hard-wired primary utility functions. However, evidence for this is both scant and complex. The apparent utilities of two likely primary events—pleasure derived from sweet tastes and pain—are not reliably blunted in depression when measured in the laboratory.^42^ ^43^ This contrasts with richer and more complex stimuli such as pictures of facial expressions, movies and music, where blunted affective ratings and physiological responses are reliably present in the appetitive and aversive domains.^44^ Since the affective value of these more complex stimuli has to be constructed and inferred, unlike that of events with primary utility, this points to the impaired inference about value as the central driver of undervaluation in depression.

### Inferred value in depression

RL is concerned with the problem of assigning and inferring value. Specifically, it provides a set of techniques to infer the long-term primary utility, either when occupying any one particular state or when faced with a particular stimulus devoid of any intrinsic primary utility itself, such as a picture. Either of the two classes of approaches to valuation, MB and MF, could in principle underlie aberrant valuation of complex stimuli.

We first briefly examine MF valuation, which, as introduced briefly above, depends on reward prediction errors. Three types of experimental set-up in humans speak to this. First are experiments examining prediction errors per se without any requirement for learning (ie, the contingencies are fully instructed), such as the monetary incentive delay task.^45^ In general, such tasks have not yielded consistent differences between depressed and control subjects in either behavioural measures or their neural correlates in the areas most strongly associated with MF learning, such as the ventral striatum.^46–50^ Second are studies explicitly examining the kind of trial-by-trial learning described by MF valuation. While these studies have shown slightly more consistent group differences at the neural level, for instance in the ventral striatum, their interpretation is complicated both by variable results in the midbrain dopaminergic regions and often by the absence of any behavioural effects.^26^ ^51–54^ Third are studies using a probabilistic response bias task.^55^ RL modelling of this task suggested that anhedonia was not related to MF learning.^56^

In contrast, several features of MB valuation appear to be involved in depression. Cognitive theories of depression have long emphasised the importance of schemas,^57^ ^58^ which, when activated by environmental triggers, lead to negative automatic thoughts and consequent aversive feelings. From a Bayesian perspective, these schemas can be viewed as priors, and the automatic thoughts as the result of a combination of the priors with the triggering sensory events. One example of this is learned helplessness, a model of depression in which healthy animals are exposed to uncontrollable stressors and come to show a variety of depression-like behavioural anomalies in other situations.^59^ These effects can arise from prior beliefs about the achievability of desirable outcomes.^60^ One would expect that, being part of the model of the world, the prior would act within the goal-directed MB valuation system. Indeed, the behavioural effects of uncontrollable stressors depend on midline prefrontal areas^61^ known to be involved in MB reasoning.^62^

One feature that is of particular interest is emotion regulation. We suggest that it again arises chiefly in MB evaluation and might be understood in terms of meta-reasoning. Since most valuation problems are computationally demanding, the MB evaluator faces the meta-reasoning problem of how to allocate its resources efficiently—that is, how to choose which evaluative (internal) actions maximise the chances of choosing the best (external) action. For example, one could sacrifice an exhaustive search of all possible outcomes to save time by only evaluating a small number of more likely scenarios. This problem has many of the features of the original valuation problem, but differs in that in theory it only incurs computational costs and not those of the real world (eg, pain). In practice, however, this distinction does not seem to hold for humans. Since imagination of aversive events has emotionally aversive consequences, internal simulations themselves also incur some of the same costs as real-world experience. Indeed, it has been found that MB valuation is exquisitely sensitive to simulated events. Healthy subjects robustly avoid plans that involve losses.^63^ It appears that patients with depression have a deficit in this inhibition of aversive processing,^64^ ^65^ with aversive stimuli hijacking rather than inhibiting processing:^66^ This is a potential cause of the repetitive negative thoughts typical of rumination^67^—a key component of the depressive vicious circle.

## Precision and D_2_ receptors in psychosis

Having discussed aspects of valuation in depression, we now turn to a discussion of inference in schizophrenia. Specifically, we explore how various neurobiological abnormalities in schizophrenia might be characterised in computational terms, and how these characterisations might aid our understanding of the disorder. We discuss reductions in synaptic gain in higher hierarchical areas, and increased presynaptic dopaminergic availability and its consequences for tonic and phasic dopaminergic signalling in the striatum.

### Psychosis, synaptic gain and precision

What are the main cortical abnormalities in schizophrenia and what do they have in common (reviewed in detail elsewhere^68^)? One key abnormality is thought to be hypofunction of the *N*-methyl-d-aspartate receptor (NMDA-R)—a glutamate receptor with profound effects on synaptic gain (due to its prolonged opening time) and synaptic plasticity (via long-term potentiation or depression)—in both the prefrontal cortex (PFC) and hippocampus (HC). A second is the reduced synthesis of γ-aminobutyric acid (GABA) by inhibitory interneurons in PFC. A third is the hypoactivation of D_1_ receptors in PFC (we shall discuss striatal hyperactivation of D_2_ receptors in the next section).

These abnormalities could all reduce synaptic gain in PFC or HC, that is, around the top of the cortical hierarchy. Synaptic gain (or ‘short term’ synaptic plasticity^69^) refers to a multiplicative change in the influence of presynaptic input on postsynaptic responses. NMDA-R hypofunction and D_1_ receptor hypoactivity are most easily related to a change in synaptic gain. Similarly, a GABAergic deficit might cause a loss of ‘synchronous’ gain. Sustained oscillations in neuronal populations are facilitated through their rhythmic inhibition by GABAergic interneurons, putatively increasing communication between neurons that oscillate in phase with each other.^70^

How can synaptic gain (and its loss) be understood in computational terms? One answer rests on the idea that the brain approximates and simplifies Bayesian inference by using probability distributions that can be encoded by a few ‘sufficient statistics’, for example, the mean and its precision (or inverse variance). While precision determines the influence one piece of information has over another in Bayesian inference, synaptic gain determines the influence one neural population has over another in neural message passing. The neurobiological substrate of precision could therefore be synaptic gain,^22^ and a loss of synaptic gain in a given area could reduce the precision of information encoded there.

**Figure 2:**
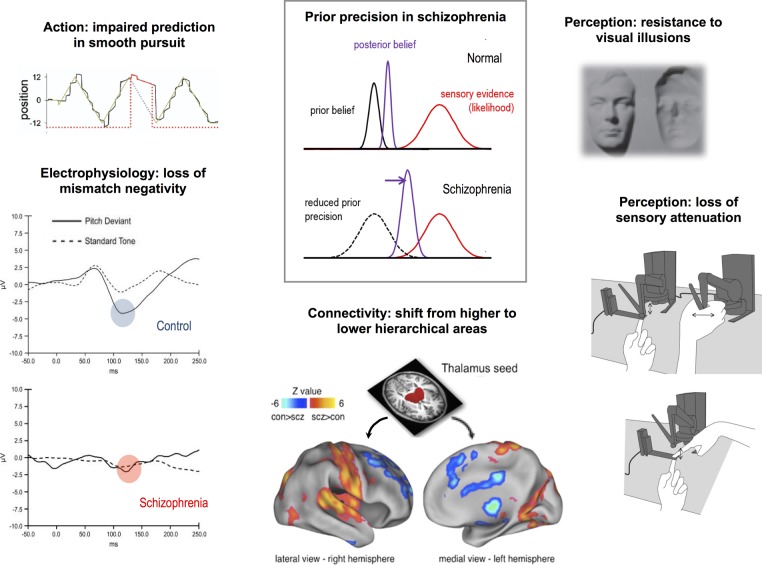
Effects of a hierarchical precision imbalance in schizophrenia. A loss of precision encoding in higher hierarchical areas would bias inference away from prior beliefs and towards sensory evidence (the likelihood), illustrated schematically in the middle panel. This single change could manifest in many ways (moving anticlockwise from left to right). (i) A loss of the ability to smoothly pursue a target moving predictably (in this plot, the patient with schizophrenia constantly falls behind the target in his eye tracking, and has to saccade to catch up again); when the target is briefly stabilised on his retina (to reveal the purely predictive element of pursuit), shown as the red unbroken line, his/her eye velocity drops very significantly (figure adapted from Hong *et al*^76^). (ii) These graphs illustrate averaged electrophysiological responses in a mismatch negativity paradigm, in which a series of identical tones is followed by a deviant (oddball) tone; in the control subject, the oddball causes a pronounced negative deflection at around 120 ms (blue circle), but in a patient with schizophrenia, there is no such deflection (red circle); that is, the brain responses to predictable and unpredictable stimuli are very similar (figure adapted from Turetsky *et al*^71^). (iii) The physiological change underlying the precision imbalance is a relative decrease in synaptic gain in high hierarchical areas, and a relative increase in lower hierarchical areas. This change would also manifest as an alteration in connectivity, shown here as significant whole brain differences in connectivity with a thalamic seed between controls and patients with schizophrenia; red/yellow areas are more strongly coupled in those with schizophrenia, and include primary sensory areas (auditory, visual, motor and somatosensory); blue areas are more weakly coupled, and include higher hierarchical areas (medial and lateral prefrontal cortex, cingulate cortex and hippocampus) and the striatum (figure adapted from Anticevic *et al*^72^). (iv) An imbalance in hierarchical precision may lead to a failure to attenuate the sensory consequences of one's own actions,^75^ here illustrated by the force-matching paradigm used to measure this effect. In this paradigm, the participant must match a target force by either pressing on a bar with their finger (below) or using a mechanical transducer (top): Control subjects tend to exert more force than necessary in the former condition, but patient with schizophrenia do not (figure adapted from Pareés *et al*^119^). (v) A loss of the precision of prior beliefs can cause a resistance to visual illusions that rely on those prior beliefs for their perceptual effects. Control subjects perceive the face on the right as a convex face lit from below, due to a powerful prior belief that faces are convex, whereas patients with schizophrenia tend to perceive it veridically as a concave (hollow) face lit from above.

A loss of synaptic gain in PFC or HC would diminish their influence over lower level areas. In the model, this would correspond to a loss of influence (ie, precision) of the model's more abstract priors over the more concrete sensory data. To the extent to which the higher levels extract and represent more stable, general features of the world, their loss might make the world look less predictable and more surprising. This simple computational change can describe a great variety of phenomena in schizophrenia (figure 2; more references and simulations of some of these phenomena are elsewhere^68^):

- At a neurophysiological level, responses to predictable stimuli resemble responses to unpredicted stimuli, and vice versa, in both perceptual electrophysiology experiments (eg, the P50 or P300 responses to tones^71^) and cognitive functional MRI paradigms;
- At a network level, higher regions of the cortex (ie, PFC and HC) have diminished connectivity to the thalamus relative to controls, whereas primary sensory areas are coupled more strongly with this region;^72^
- At a perceptual level, a greater resistance to visual illusions^73^ (which exploit the effects of visual priors on ambiguous images, eg, the famous ‘hollow-mask’ illusion^74^) and a failure to attenuate the sensory consequences of one's own actions, which could diminish one's sense of agency;^75^
- At a behavioural level, impaired smooth visual pursuit of a predictably moving target, but better tracking of a sudden unpredictable change in a target's motion.^76^

An alternative interpretation of these changes is that pathology in the PFC or HC (eg, in postsynaptic signalling and neurotrophic pathways^77^) might impair the formation and representation of prior beliefs more generally, rather than directly and selectively affecting only a separate representation of their certainty. Indeed, if this were the case, then reducing the influence of aberrant beliefs on sensory processing might even be computationally adaptive (and physiological rather than pathophysiological), since it would reduce their (possibly misleading) influence on inference.

How do the above ideas relate to the actual symptoms of psychosis? A reasonable hypothesis would be that a loss of high-level precision in the brain's hierarchical inference might result in diffuse, generalised cognitive problems (as are routinely found in schizophrenia^78^) and overattention to sensory stimuli (as is found in the ‘delusional mood’; similar in some respects to the loss of central coherence in autism—see below). In addition, it could lead to the formation of more specific unusual beliefs, as the reduced high-level precision permits updates to beliefs that are larger and less constrained. This is because the precision of (low-level) prediction errors is much higher, relative to the (high-level) prior beliefs (an imbalance reflected in connectivity analyses^72^). However, one might expect that these unusual beliefs should be fleeting—as they themselves would be vulnerable to rapid updating—unlike delusions.

This account raises two important (and, we propose, related) questions that we address in the next section. First, if high-level precision is generally low, why do delusions, which appear to exist at a reasonably high (conceptual) level in the hierarchy, become so fixed? Second, what is the computational impact of the best-established neurobiological abnormality in schizophrenia—an elevation in presynaptic dopamine?^79^

### Striatal presynaptic dopamine elevation

The positive symptoms of schizophrenia are strongly associated with the elevated presynaptic availability of dopamine in the dorsal (associative) striatum,^79^ and are reduced by D_2_ receptor (but not D_1_ receptor) antagonists (although neither is always the case^80^). Increased stimulation of striatal D_2_ receptors might then be a sufficient cause of psychosis, but the exact nature of this stimulation and how it causes psychotic symptoms remains unclear.

In electrophysiological studies, dopamine neurons show both tonic and phasic firing patterns;^81^ D_1_ receptors are most sensitive to phasic bursts, whereas tonic activity and phasic pauses are best detected by D_2_ receptors.^82^ These patterns cannot be distinguished using brain imaging in humans, unless their computational roles can be modelled and thus their quantities inferred from behaviour. It is as yet unclear how the increased presynaptic availability of dopamine alters these patterns in schizophrenia: One might expect that both tonic and phasic release would increase in proportion, but an increase in tonic release could reduce phasic release, for example, via inhibitory presynaptic receptors,^83^ and these two modes of release have also been argued to be at least partially independent.^81^ We now explore how these patterns may be disrupted in schizophrenia, and how this might affect computations.

#### Tonic dopamine signalling

Tonic striatal D_2_ hyperstimulation is thought to increase inhibition of the corticostriatothalamocortical loops in the so-called indirect pathway through the striatum. The indirect pathway itself contains two inhibitory pathways. One (via the subthalamic nucleus) causes blanket inhibition of action and acts as a brake, but the other is channelised^84^ such that it can help switching to alternative actions.

If the indirect pathway enables switching, then increased tonic D_2_ receptor activity in the striatum ought to oppose this (interestingly, D_2_ receptors also suppress alternative task ‘rules’ in PFC^85^). Indeed, reversal learning performance decreases with increasing occupancy of D_2_ receptors in the dorsal^86^ ^87^ and ventral^81^ striatum, and with genetic variants in the dopamine transporter that might increase tonic dopamine.^88^ Sufferers of schizophrenia are impaired at reversal learning over and above their generalised cognitive impairment,^89^ ^90^ which is in keeping with these findings. D_2_-mediated inflexibility might even make delusions so resistant to change.

From a computational point of view, this striatal D_2_ hyperstimulation could reduce an agent's perception of volatility in the world (x_3_ in figure 1), causing action tendencies (known as policies) to become more fixed and have (incorrectly) high ‘precision’. Indeed, work in addiction has similarly argued that dopamine promotes rather less flexible habits over goal-directed choices.^91^ This is interesting because it is conceivable that a hyperdopaminergic increase in (dorsal striatal) precision of policies might occur as an adaptation to a loss of (prefrontal) high-level precision (ω_2_ in figure 1),^68^ that is, that the excessive dorsal striatal dopamine release found in prefrontal dysfunction^92^ ^93^ is an attempt to stabilise thoughts and action selection in the face of cognitive instability.

Even if this is so, one must still ask why so many other risk factors for schizophrenia—for example, social isolation or subordination, prenatal or perinatal adversity, and acute stress—cause dopamine hyperactivity?^79^ One could argue that the computational commonality among these factors is an increase in predicted environmental volatility, but raised tonic dopamine release makes decisions less, not more, volatile.

#### Phasic dopamine signalling

The phasic responses of dopamine neurons comprise bursts and pauses, which facilitate the excitatory ‘direct’ pathway through excitatory D_1_ receptors, and facilitate the inhibitory ‘indirect’ pathway through inhibitory D_2_ receptors, respectively. These have been proposed to reflect reward prediction errors^94^ (but also aversive prediction errors^95^ and the precision or ‘salience’ of prediction errors^96^) in the ventral and dorsal striatum. Actor-critic models^6^ propose that reward prediction errors act as signals which teach the ventral striatum (critic) the values of states, and the dorsal striatum (actor) to associate states with optimal actions (by increasing the excitation or reducing the inhibition of the currently selected action^6^), with recent evidence for a causal role of dopamine in this regard.^97^

In fMRI paradigms designed to elicit reward prediction error and reward prediction signals, patients with schizophrenia show diminished appropriate activations^98^ but greater inappropriate activations in the ventral striatum,^99^ compared with controls. Similar patterns were observed in an associative learning task without explicit rewards;^100^ however, fMRI cannot tell whether these abnormalities are due to abnormal phasic dopamine signalling. Although behavioural responses in such tasks are often less abnormal than the underlying neural activity,^101^ computational modelling of behaviour in the beads task^102^ and reward learning tasks^27^ suggests that the impact of phasic positive feedback is diminished in schizophrenia. This may indicate that an elevated tonic dopamine level is reducing phasic bursts (reward learning) but not phasic pauses (punishment learning), and indeed PET studies suggest an inverse relationship between tonic DA and phasic BOLD signals.^103^ An alternative suggestion is that this apparent reward learning deficit may actually be caused by reduced working memory capacity.^104^

#### Incentive (and aberrant) salience

The theory of incentive salience proposes that ventral striatal dopamine signalling (whether phasic or tonic) gives motivational impetus to act on stimuli whose values have already been learnt.^105^ Incentive salience is closely related to MF learning of values,^91^ ^106^ and might also be related to the precision or confidence of beliefs that actions will have preferred outcomes.^96^ In the ‘aberrant salience’ hypothesis, Kapur^107^ proposed that there is aberrant (ie, increased inappropriate) signalling of incentive salience in patients with positive psychotic symptoms. Unmedicated prodromal psychotic patients do experience—in proportion to their positive symptoms—irrelevant features of stimuli in a reward learning task as ‘aberrantly salient’ (although this is not obviously reflected in their reaction times), but their striatal activations are harder to interpret.^108^

A weakness of the aberrant salience hypothesis is that the connection between aberrant motivational signalling and the abnormal inference (hallucinations) and abnormal learning (delusions) found in positive symptoms is not intuitive (assuming that delusions do indeed involve abnormal learning). One could also argue that aberrant motivational salience works best as an account of manic psychosis—in which the patient is energised and perceives events in a positive light—rather than schizophrenic psychosis, which is often aversive in nature. Conversely, diminished appropriate salience signalling (not part of the original hypothesis but identified in several studies) provides a plausible explanation for negative symptoms; and, indeed, a loss of ventral striatal activation to rewards has been shown to be proportional to negative symptoms in unmedicated patients with schizophrenia.^109^

Aside from aberrant salience, there are many other potential explanations for negative symptoms;^15^ for example, pronounced asymmetry in learning (ie, a failure to learn stimulus-reward associations but intact learning of stimulus-punishment associations), a failure to infer the values of actions (cf. anhedonia in depression), greater discounting of rewards that require effort,^110^ and a loss of uncertainty-driven exploration such that valuable states are never discovered.

## Conclusion

In this brief overview, we have attempted to highlight some aspects of the Computational Psychiatry approach to characterising and measuring the brain's inferences. We have not had space to review Computational Psychiatry approaches to many mental disorders, such as anxiety,^111^ personality disorder,^8^ autism,^112^ attention deficit hyperactivity disorder,^113^ addiction,^6^ functional symptoms^114^ and others. We focused instead on two examples: The first was that of depression, where we suggested that MB valuation may be the at the root of anhedonia.^27^ Our second example was the concept of reduced high-level precision in schizophrenia. We must emphasise, however, that their description in the context of specific disorders should not necessarily be taken to imply specificity to these disorders. The dysfunctions in meta-reasoning described here in depression could probably be applied equally well to certain anxiety disorders.^115^ Anhedonia is also found in schizophrenia, and in this context just as in depression, hedonic responses to primary rewards seem normal,^116^ yet pleasure-seeking behaviour is reduced. If so, Computational Psychiatry might help identify transdiagnostic computational mechanisms: we must then investigate the extent to which such mechanisms share biological substrates.

Similarly, alterations in the use or representation of uncertainty may underlie phenomena not just in schizophrenia, but also in autism (eg, a resistance to visual illusions, and sensory overattention^112^). However, an important difference between the disorders may be that this reduction in precision arises earlier in development in autism. Thus, while sufferers of both disorders may have uncertainty about the mental states of others, those with autism have never learnt to attribute mental states to others. In contrast, those with schizophrenia have done so in the past, but in the present they find themselves in a state of high anxiety and uncertain of others’ intentions, possibly suggesting why paranoid persecutory ideas might be commonly found in schizophrenia, but not autism. In this case, then, common computational mechanisms may undergo distinct interactions with the environment. In both cases, the hope is that Computational Psychiatry will identify patterns that map more closely onto the underlying neurobiology than current diagnoses do.

Aside from disorders that are thought of as purely psychiatric, Computational Psychiatry has been used to examine putatively dopaminergic computations in neurological conditions such as Parkinson's disease and Tourette's syndrome. In some classic studies, Frank and colleagues devised a probabilistic task which quantifies individuals’ abilities to learn from positive and negative feedback, encoded by dopamine bursts and pauses, respectively. They showed that dopamine-depleted patients with Parkinson's disease were better than controls at learning from negative feedback, but dopaminergic medication reverses this bias.^6^

This asymmetry could underlie the phenomenon of pathological gambling in the context of dopamine agonist treatment: such patients may be able to learn only from their wins but not their losses. Interestingly, the opposite asymmetry (better learning from positive feedback, reversed by antidopaminergic medication) was seen in the hyperdopaminergic Tourette's syndrome.^117^ Computational Psychiatry approaches are also being used to investigate motivation and effort cost in apathy^118^—found in Parkinson's disease and numerous other neurological conditions—and it will be interesting to see how many underlying computational mechanisms are shared with negative symptoms in schizophrenia.

Computational Psychiatry is no panacea, however, and several important pitfalls ought to be mentioned. The first is the interpretation of the results of Bayesian model selection: the ‘best’ model is extremely unlikely to model the true generative process correctly, and may be but the best of a set of bad models. Model comparison must always be accompanied by model validation, which involves generating surrogate data from the model and comparing it qualitatively with the data of interest.

Generating data will in fact often identify systematic failures of models that may confound the interpretation of their parameters. This will require the addition of ad hoc parameters to explain specific aspects of the data that may interfere with inference, but not be of interest. Consider a data set where a patient has an idiosyncratic preference for responding to the left or to the right. If such a preference is not allowed for in the model, then the parameters of the model will be forced to explain such a preference. This may lead to spurious conclusions, just as the failure to recognise such irrelevant idiosyncrasies may confound classical analyses.

Furthermore, modelling does not replace careful experimental design. If a task does not exploit a particular computation, then that computation cannot be examined simply by fitting a model requiring that computation to the data. Put differently, the complexity of the model must be supported by complex data, and this in turn requires appropriate experimental design. The last pitfall we shall mention concerns a ubiquitous parameter in decision-making models: the ‘temperature’ parameter of the softmax action selection function. This controls the stochasticity of decision-making, such that the greater it is, the less decisions reflect the values of different options, that is, the more random they are. It is always possible, however, that a difference in the stochasticity of responses between groups reflects a non-random process that has not been included in the model. Hence, it is important to examine whether the noise assumed by the model actually matches that observed in the data.

Our fundamental message is that thinking of the brain as having to solve inferential problems can be a fruitful way of generating testable computational hypotheses about psychiatric disorders. The Bayesian perspective formalises the key aspects of inference and underlines the importance of uncertainty, while the RL view formalises the key aspects of choice. Characterising psychiatric disorders as problems of inference or learning—whether in the domain of rewards, threats, somatic percepts, ‘external’ percepts or social inferences—makes them tractable to analysis with these techniques.

## Footnotes

i In mathematical terms, discriminative models learn p(causes|data)—the probability of some causes, given the data—whereas generative models learn the reverse, p(data|causes), and use that (and p(causes) and Bayes’ theorem) to compute p(causes|data).
